# Golgi-Cox Staining Protocol for Medium Spiny Neurons in the Striatum of Neonatal and Early Postnatal Mouse Brain Using Cryosections

**DOI:** 10.3390/ijms26167870

**Published:** 2025-08-14

**Authors:** Heba A. Ali, Wafaa Mahmoud, Jihad A. M. Alzyoud, Iman Aolymat, Saad AL-Nassan

**Affiliations:** 1 Department of Basic Dental Sciences, Faculty of Dentistry, The Hashemite University, P.O. Box 330127, Zarqa 13133, Jordan; jihada@hu.edu.jo; 2 Department of Anatomy, Faculty of Medicine, Jordan University of Science and Technology, P.O. Box 3030, Irbid 22110, Jordan; washunnaq@just.edu.jo; 3 Department of Anatomy, Physiology and Biochemistry, Faculty of Medicine, The Hashemite University, P.O. Box 330127, Zarqa 13133, Jordan; imank@hu.edu.jo; 4 Department of Physiotherapy, Faculty of Allied Health Sciences, Al-Ahliyya Amman University, P.O. Box 19111, Amman 19328, Jordan; saad@hu.edu.jo; 5 Department of Physiotherapy, Faculty of Applied Medical Sciences, The Hashemite University, P.O. Box 330127, Zarqa 13133, Jordan

**Keywords:** Golgi-Cox, neonatal, postnatal, mouse brain, medium spiny neurons, dendritic spines, striatum, cryosections, tissue integrity, neurodevelopment

## Abstract

Studying the morphological changes in dendrites and dendritic spines during the early postnatal period is essential for unraveling the development of neural circuits and synaptic connectivity. Structural alterations in the dendritic arborization and spine morphology of medium spiny neurons (MSNs) have been closely linked to various neurodevelopmental disorders (NDDs). While Golgi-Cox staining remains a powerful technique for visualizing individual neurons, existing protocols are predominantly optimized for adult rodent brains only. This has limited our insight into MSNs development during the early postnatal stages, largely due to difficulties in maintaining tissue integrity during processing and the absence of standardized methods specific to neonatal brains. In this study, we present a reliable, cost-effective, and easily reproducible Golgi-Cox staining protocol suitable for use in standard histology laboratories. This protocol is specifically adapted for neonatal and early postnatal mouse brain tissue but is also applicable to adult brains. It enables consistent and detailed analysis of dendritic and spine morphology across developmental time points and provides a valuable tool for investigating the disrupted neuronal maturation observed in the mouse models of NDDs.

## 1. Introduction

Following the completion of neuronal formation shortly after birth, the mouse striatum undergoes rapid maturation with a marked increase in the glutamatergic corticostriatal and dopaminergic nigrostriatal input between the first and fourth weeks of age [1,2]. During this developmental window, dendritic branching and spine formation increase considerably to establish functional neural circuits. Early postnatal weeks in rodents represent a critical period for striatal development during which significant events and rapid morphological changes take place [3,4]. Dendrites and spines morphology are key determinants of the connectivity and maturation status of the excitatory and inhibitory striatal connections.

Environmental modifications and sensory deprivation during early postnatal development have been associated with abnormal dendritic spine development. For instance, socially isolating mice by housing them individually away from their littermates at three weeks of age has been associated with the formation of thinner and smaller dendritic spines in the prefrontal cortex during adulthood [5]. Also, a notable increase in dendritic spine density on the distal apical branches of pyramidal neurons was reported in lead-exposed kittens during the postnatal period [6]. Moreover, another study demonstrated that prolonged sensory deprivation—achieved by whisker trimming in three-week-old mice—resulted in reduced dendritic spine elimination in the primary somatosensory cortex [7].

In genetically modified mouse models of NDDs, changes in dendritic structure and spine morphology have been linked to significant motor and behavioral abnormalities in adulthood [8]. However, behavioral impairments often emerge as early as the second or third postnatal week in many of these models [9], indicating that disruptions in dendritic growth and spine formation may begin during, or even before, this critical period of brain development. For instance, decreased synaptic activity has been observed as early as the first postnatal week in a Huntington’s disease mouse model, and initiating treatment during this neonatal window may help mitigate adult symptoms [10,11]. Studying neurons during early postnatal development, particularly from postnatal day 0 (PND0) to postnatal day 30 (PND30), is important, since it marks the essential phase of synaptic development and neuronal circuit maturation. Postnatal stages are therefore crucial, particularly in knockout and transgenic models with high mortality, where adult brain analysis is not possible [12,13]. Consequently, delaying the examination of neuronal morphology until adulthood, as seen in many existing studies, may miss important early neurodevelopmental changes.

Golgi staining, which was first described by Camillo Golgi [14], is a fundamental method to clearly visualize the detailed structure of individual neurons, including the soma and dendritic tree. The Golgi-Cox method is a modified staining technique that uses mercuric chloride after 14 days of impregnation. Most published Golgi-Cox protocols and their modifications were optimized to be used in adult or embryonic brain tissue, but not early postnatal tissue [15,16,17,18,19,20]. Other studies reported that the use of Golgi-Cox in neonatal and early postnatal mouse brains have mainly used expensive commercially available rapid Golgi-Cox kits [20,21,22]. In [23], the development of the rat superior colliculus was examined across various postnatal stages from day 3 to 45. This study employed a prolonged Golgi impregnation period of 4 to 5 weeks, lacked clear immunohistochemical visualization of the stained neurons, and mainly focused on dendritic tree morphology without detailed analysis of dendritic spines’ structural features. Neonatal and early postnatal brains are fragile and prone to damage more easily compared to adult brains during dissecting the brains out of the skull and histological preparation steps. This is attributed to the higher water content and reduced myelination in the immature postnatal brain [24,25]. In addition, Golgi-Cox impregnation makes the tissue increasingly fragile after 14 days, which significantly complicates obtaining high-quality sections without tearing or cracking, thereby posing a challenge for reliable microscopic analysis.

Despite numerous modifications to the Golgi-Cox staining technique, a standardized method suitable for consistently tracking dendritic and spine maturation across various early neonatal and postnatal stages remains lacking. In this study, we present an efficient, cost-effective, and reproducible Golgi-Cox staining protocol for neonatal and early postnatal mouse brains, aged between PND1–PND30. This method preserves tissue integrity, enabling detailed and uninterrupted visualization of MSNs in the striatum using 100 μm thick cryosections. Importantly, it utilizes affordable reagents without the need for fixatives or expensive commercially available kits and can be implemented in any standard histology laboratories. This enables more in-depth neurological investigations during the earliest stages of brain development, which is crucial, as disruptions in this process are frequently associated with NDDs.

## 2. Results

### 2.1. Optimization of Cryoprotection Step with Golgi-Impregnated Neonatal, Early Postnatal, and Adult Mouse Brain

**Figure 1:**
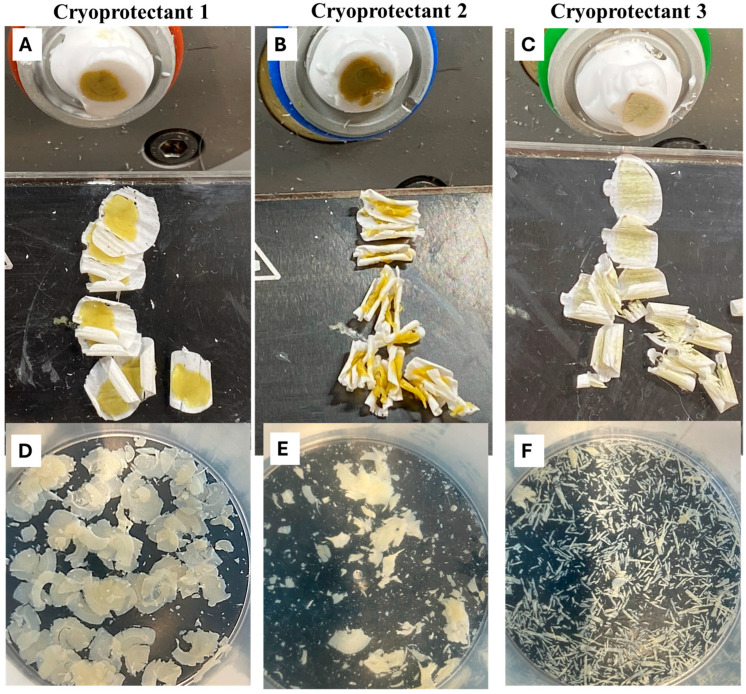
Comparing structural preservation after using three cryoprotectant solutions for Cryostat Sectioning of Golgi-Cox-impregnated mouse brains. (**A**) cryoprotectant 1 resulted in intact and undistorted brain sections. (**B**) Cryoprotectant 2 left brain sections overly sticky and torn. (**C**) Cryoprotectant 3, 25% sucrose made sections very porous and cracked. Images of adult brain sections inside 50 mL plastic containers filled with 30 mL PBS reveal that brain sections cryoprotected using solution 1 were collected in PBS with preservation of tissue integrity (**D**), while it was difficult to collect tissue treated with cryoprotectant solution 2 (**E**) and cryoprotectant solution 3 (**F**).

To determine the optimal cryoprotectant solution for this study, we tested several formulations previously described in the literature. Among these, cryoprotectant 1, modified from [16] and consisting of 25% sucrose and 15% glycerol in Phosphate buffered Saline (PBS), proved to be the most effective one. It provided excellent structural preservation and was optimal for sectioning of both postnatal and adult brain tissue (Figure 1A). In contrast, cryoprotectant 2, described in [26], was previously used successfully with adult rat tissue at a thickness of 200 µm has resulted in overly sticky tissue that was unsuitable for sectioning in our work (Figure 1B). Cryoprotectant 3 rendered the tissue porous and excessively rigid and resulted in significant tissue cracking (Figure 1C). When attempting to collect brain sections, only adult Golgi-impregnated tissue cryoprotected with the sucrose and glycerol solution (Cryoprotectant 1) could be successfully collected and temporarily stored in PBS at 4 °C for later mounting on microscopic slides without compromising tissue integrity (Figure 1D). This feature offered greater flexibility in workflow and convenience for researchers processing large numbers of mouse brains, allowing the procedure to be carried out over multiple days without compromising tissue integrity. Notably, adult brain tissue remained stable in PBS at 4 °C for 3–7 days without significant degradation. In contrast, tissues cryoprotected with cryoprotectant 2 (sucrose with ethylene glycol) and cryoprotectant 3 (25% sucrose alone) were notably unstable following sectioning. These sections exhibited significant disintegration and fragmentation during PBS storage, rendering them unusable for mounting and analysis (Figure 1E,F).

### 2.2. Golgi-Cox Staining Quality and Tissue Integrity Across All Age Groups

**Figure 2:**
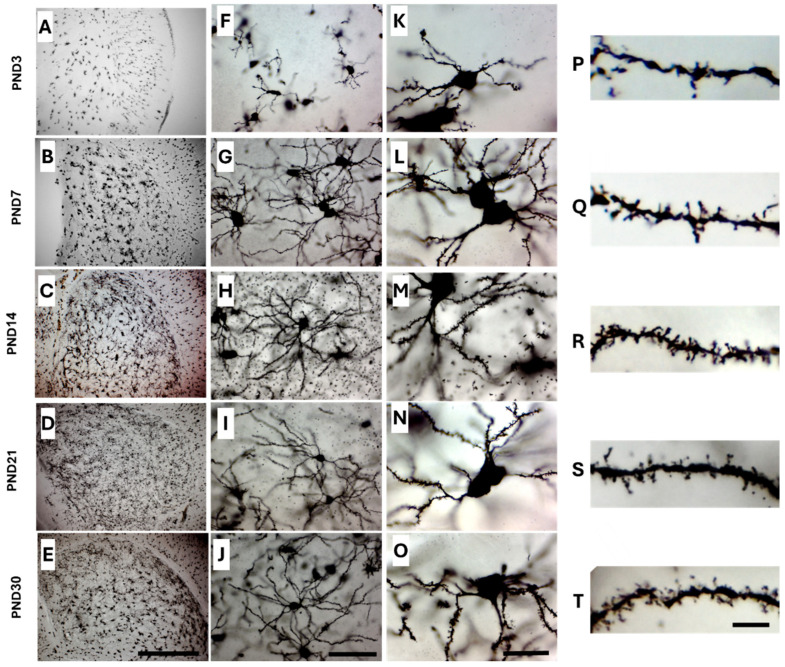
Representative images of Golgi-Cox-impregnated brain sections taken at different magnifications 40× (**A**–**E**), 400× (**F**–**J**), and 1000× (**K**–**T**) from different postnatal developmental groups; PND3, PND7, PND14, PND21, and PND30. (**A**–**E**) show consistent staining of MSNs, with well-preserved tissue sections. (**F**–**J**) display well -defined cell bodies and fully extended dendritic trees. At higher magnification (**K**–**O**), cell bodies and dendritic spines exhibit strong contrast and clearly defined. (**P**–**T**) show dendritic spines as small protrusions along the dendritic branches, with consistent staining throughout the entire dendrite segment enabling detailed analysis of dendritic spine morphology across all age groups. All brains were immersed in Golgi-Cox solution for 14 days for impregnation without prior fixation. Scale bars: 500 µm in (**E**), 50 µm in (**J**,**O**), and 5 µm in (**T**).

Tissue integrity and structural preservation of mouse brain sections were well-preserved throughout the Golgi-Cox staining procedure across all tested age groups, indicating that the protocol did not compromise the morphological quality of the samples (Figure 2).

The quality of Golgi-Cox staining was assessed qualitatively by examining the morphology of MSNs under light microscopy. Brain sections from different age groups showed a consistent and high-quality Golgi-Cox-stained MSNs (Figure 2A–E), allowing detailed visualization of neuronal structures, including dendrites, cell soma, and dendritic spines in all age groups. Neurons showed consistent staining quality, with well-preserved cell bodies and fully extended dendritic trees (Figure 2F–J). At higher magnification, neuronal cell bodies of Golgi-Cox-stained MSNs appear intact, well-defined, and clearly distinguishable, with visible primary dendrites and minimal background staining (Figure 2K–O). Dendritic spines are clearly and consistently stained along the dendritic branches, enabling detailed analysis of dendritic spine morphology across all age groups (Figure 2P–T). 

### 2.3. Optimizing Golgi-Cox Staining for Neonatal Mouse Brain (PND1 and PND3)

**Figure 3:**
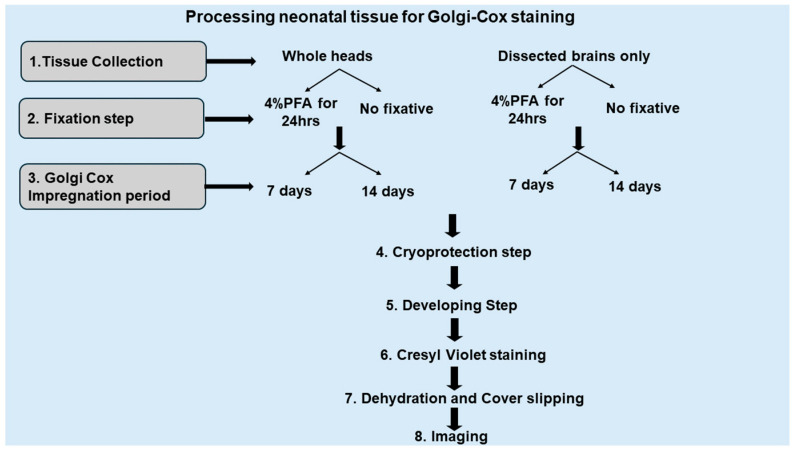
Experimental plan for optimizing Golgi-Cox staining of neonatal mouse MSNs.

The Golgi-Cox staining protocol described here was specifically optimized for the neonatal age groups examined in this study (PND1 and PND3), as detailed in the experimental design shown in Figure 3. The aim was to determine whether the number of stained neurons could be increased at these two time points while maintaining high staining quality.

#### 2.3.1. Comparison Between Impregnated Dissected Brains and Whole Heads

No observable differences were found between non-fixed and 14-days Golgi–cox-impregnated PND1 and PND3 dissected brains and whole heads following Golgi-Cox impregnation. Both preparations showed comparable levels of staining quality, tissue preservation, and neuronal detail. Morphological features, including dendritic arborization and spine visibility, were equally well-impregnated in both groups, suggesting that whole-head immersion did not compromise staining efficacy compared to dissected brain samples.

#### 2.3.2. Combining Golgi-Cox and Nissl Staining Enhances Demarcation of Striatal Boundaries and Visualization of Individual Neurons in the Neonatal Brain

**Figure 4:**
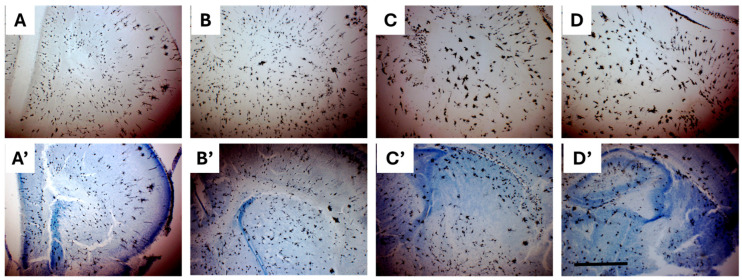
Representative Golgi-Cox-impregnated coronal sections from PND1 and PND3 mouse brains. (**A**–**D**): Golgi-Cox staining reveals neuronal morphology but does not clearly define certain brain region boundaries. (**A′**–**D′**): The same sections counterstained with cresyl violet, enhancing cytoarchitectural contrast and enabling precise delineation of anatomical boundaries. Scale bar: 1000 µm.

Nissl staining is routinely used to visualize cytoarchitecture and accurately delineate regional neuronal boundaries. Since Golgi-Cox staining selectively labels a random subset of neurons, it was challenging to demarcate anatomical boundaries between regions such as the striatum and adjacent structures in the immature PND1 and PND3 brain sections using this method alone (Figure 4A–D). This limitation is primarily due to the incomplete structural development and maturation present at these early neonatal stages. To address this, Golgi-Cox staining was followed by Cresyl Violet staining on the same tissue sections, achieved by removing the coverslips from previously stained slides to enhance cytoarchitectural contrast and enabling more precise identification of anatomical boundaries (Figure 4A′–D′). This combined staining method was applied exclusively to neonatal tissue, where structural development is still in its early stages.

#### 2.3.3. An Impregnation Time of 14 Days Is Required for Both Adult and Neonatal Tissue

**Figure 5:**
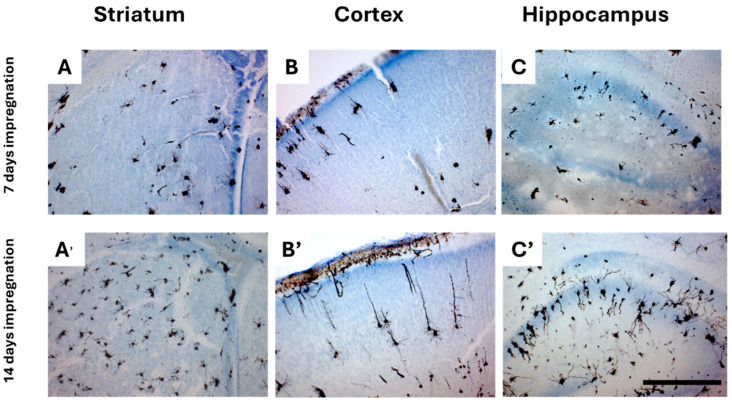
Effect of impregnation duration on Golgi-Cox staining quality in PND1 neonatal mouse brain sections. Representative coronal brain sections at 100× magnification showing Golgi-Cox staining after two different impregnation periods, 7 days (**A**–**C**) and 14 days (**A′**–**C′**). Shortened impregnation time (7 days) resulted in a noticeably reduced number of positively stained neurons, weaker staining, and fewer labeled neurons. Scale bar: 500 µm.

Given the smaller size and higher permeability of neonatal brain tissue, we evaluated two different impregnation periods for the PND1 and PND3 mouse brains to assess whether a shorter impregnation period could produce staining quality comparable to the standard 14-day protocol used for the subsequent age groups studied in this paper. Our results showed that reducing the Golgi-Cox impregnation time resulted in a lower number of stained neurons (Figure 5A–C) compared to the 14-day impregnation, which yielded more extensive staining across multiple brain regions, including the striatum, cortex, and hippocampus (Figure 5A′–C′).

#### 2.3.4. Fixation with 4% Paraformaldehyde (PFA) vs. Non-Fixation

**Figure 6:**
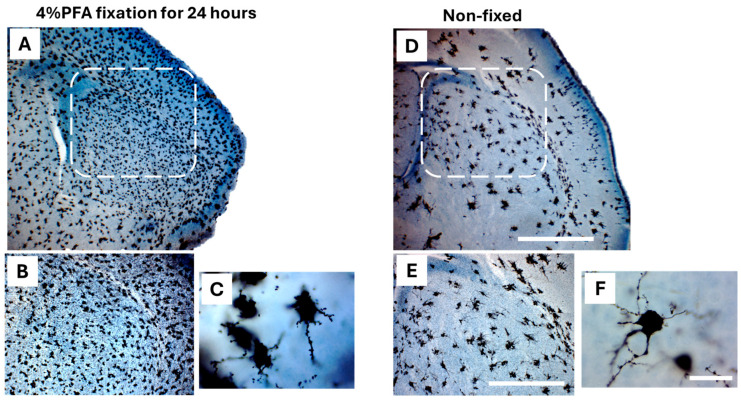
Effect of 4% PFA fixation on Golgi-Cox staining quality in PND1 mouse brain. Representative coronal sections of PND1 mouse brains stained with the Golgi-Cox method to compare tissues pre-fixed with 4% PFA (**A**–**C**) to tissue without pre-fixation (**D**–**F**). PFA fixation (**A**,**B**, left side) resulted in a noticeable increase in the number of Golgi-Cox-impregnated neurons and improved overall staining intensity compared to non-fixed tissue (**C**,**E**, right side). However, at higher magnification PFA-fixed tissue in (**C**) showed reduced clarity in soma delineation and incomplete dendritic staining compared to non-fixed tissue (**F**). Scale bars: 1000 µm in (**D**), 500 µm in (**E**), and 50 µm in (**F**).

To see if fixation of the neonatal brains with 4% PFA would enhance the number and intensity of Golgi-Cox-stained neurons. PND1 and PND3 brains were fixed in 4%(PFA) for 24 h prior to Golgi-Cox processing, followed by 14 days of impregnation in Golgi-Cox. Notably, this pre-fixation step improved the handling of delicate neonatal tissue and made the mounting step easier. It has also reduced brittleness, minimized section loss during processing, and increased the number of Golgi-Cox-impregnated neurons. However, when examining brain sections under the microscope it resulted in densely impregnated neurons (Figure 6A,B) compared to unfixed tissue (Figure 6D,E). While at higher magnification, cell bodies were poorly delineated, and dendritic trees notably interrupted in fixed tissue (Figure 6C) compared to unfixed tissue (Figure 6F).

### 2.4. Golgi-Cox Staining Protocol Is Suitable to Use with Adult Tissue

**Figure 7:**
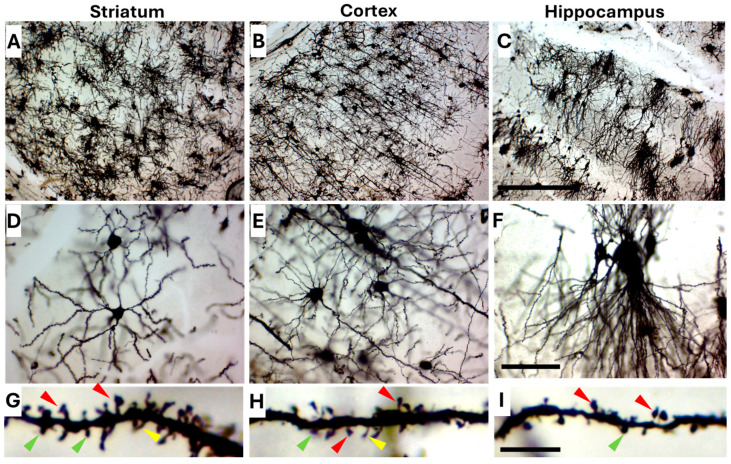
Golgi-Cox staining of neurons in adult (3-month-old) mouse brain across multiple brain regions. Representative images show Golgi-Cox staining in striatum (**A**), cortex (**B**), and hippocampus (**C**). Neurons across all regions appear well impregnated with clear contrast and minimal background. Each region is presented at 100× magnification (**A**–**C**) to illustrate overall tissue architecture and staining intensity and consistency, and at 400× magnification (**D**–**F**) to highlight neuronal morphology in detail. At higher magnification 1000× (**G**–**I**), dendritic arbors and spines are sharply defined and suitable for quantitative spine analysis. Different spine types are indicated by arrow heads, color-coded as following (red: mushroom spines, green: stubby, yellow: thin. Scale bars are 200 µm in (**C**). 50 µm in (**F**) and 5 µm (**I**).

Although the Golgi-Cox staining protocol described here was optimized for neonatal and early postnatal mouse brains, it also yielded excellent results in adult brain tissue (Figure 7). High-quality impregnation was achieved across multiple regions, including the striatum, cortex, and hippocampus, with well-preserved neuronal morphology and consistent, reproducible staining (Figure 7A–C). Neurons in all examined regions displayed strong contrast and minimal background with continuous and clear dendritic arbors (Figure 7D–F). At high magnification, individual spines were sharply resolved, enabling reliable quantitative spine analysis. Distinct spine subtypes were easily identified, as indicated by color-coded arrowheads: red for mushroom spines, green for stubby spines, and yellow for thin spines (Figure 7H,I).

**Figure 8:**
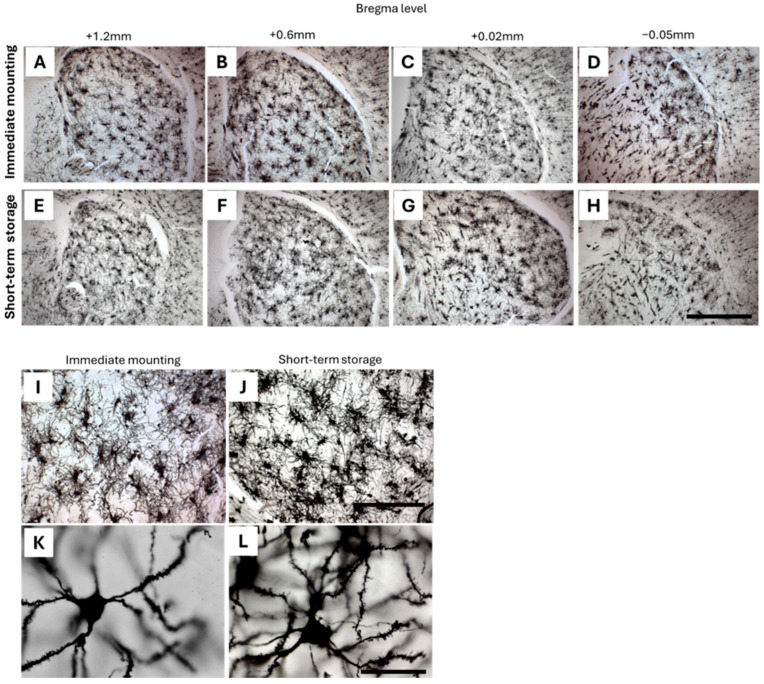
Evaluation of section integrity in adult brain tissue following immediate versus delayed mounting. Representative images of 3-month-old mouse brain sections collected at various Bregma levels are shown, either mounted immediately after cryostat sectioning (**A**–**D**) or after storage in PBS at 4 °C for up to 7 days prior to mounting (**E**–**H**), demonstrating preserved section integrity in both conditions. At higher magnifications, stored sections displayed structural preservation comparable to that of freshly mounted tissues, including intact dendritic arbors (**I**,**J**) and clearly visible dendritic spines (**K**,**L**). Scale bars: 500 µm (**H**), 200 µm (**J**), and 50 µm (**L**).

To assess the adaptability of our workflow, we compared the integrity of adult (3-month-old) brain sections mounted immediately after cryostat sectioning with those stored in PBS at 4 °C prior to mounting (Figure 8). Microscopic analysis across multiple Bregma levels, from rostral to caudal, demonstrated that sections stored for up to 7 days (Figure 8E–H) maintained structural integrity comparable to those mounted immediately (Figure 8A–D). Furthermore, stored sections exhibited well-preserved dendritic arbors and clearly visible dendritic spines (Figure 8J,L), similar to those observed in directly mounted sections (Figure 8I,K). These findings indicate that short-term storage in PBS does not compromise fine morphological details and highlight the practicality of this approach as a viable strategy during labor-intensive workflows.

## 3. Discussion

Golgi-Cox staining has long been a powerful tool for studying neuronal morphology. This method allows for detailed visualization of dendritic architecture and dendritic spines that are frequently disrupted in NDDs. While most previously published Golgi-Cox methods are optimized to study detailed neuronal structure in adult rodents’ brains only [17,26,27], little is known about the method’s applicability at early postnatal and neonatal stages. At these developmental periods, the integrity of brain structures is reduced compared to adult brains making technical processing and tissue preservation very challenging. Here we present a Golgi-Cox protocol optimized for neonatal and early postnatal mouse brains with preservation of tissue structure and clear visualization of the morphology of individual neurons. Time points selected in this research work provide a framework for the study and assessment of neurodevelopment as they correspond to key events in the formation and maturation of neural circuits in mice [3,28].

Studying neuronal morphology during early postnatal development of mouse striatum is essential, as this period marks the onset of neural circuit development and maturation. In mice, neural circuits connecting cortex and thalamus with striatum begin to develop in late embryonic stages and continue during early postnatal weeks [9,29]. Furthermore, many transgenic mouse models of NDDs manifest their behavioral deficits early during the second and third postnatal weeks [9,30,31], suggesting that changes in dendritic tree and dendritic spines might have started during or before this developmental stage. Criteria for successful Golgi-Cox staining include clear and consistent staining of dendritic arbors and dendritic spines with minimum background. Tissue tearing and cracking during the processing and cutting of Golgi-Cox-impregnated neurons can significantly render the achievement of analysis, particularly those of dendritic tree analysis. Therefore, optimizing handling and cutting techniques is essential to preserve structural integrity and ensure accurate assessment of dendritic architecture and spine morphology.

The methodology described in this study is specifically applied to Golgi-Cox-impregnated neonatal and early postnatal mouse brains, which are very delicate and immature—particularly those from PND1 and PND3 mice. Two key methodological strategies were instrumental in achieving reliable and high-quality staining: (1) the use of cryostat sectioning and (2) the decision to omit pre-fixation with PFA prior to Golgi-Cox impregnation.

Neonatal brain tissue presents distinct anatomical and mechanical challenges. High water content, immature myelination, and underdeveloped structural integrity combined with the inherent brittleness of unfixed, Golgi-Cox-impregnated tissue render early postnatal brains particularly vulnerable to mechanical distortion during processing [24,25]. While vibratome sectioning is widely used for thicker sections in adult brains [32], our observations revealed that it is not well-suited to sectioning fragile, unfixed neonatal tissue. The pressure applied during cutting frequently might cause compression, tissue tearing, and loss of morphological detail. Although both cryostat and vibratome sectioning methods are capable of producing 100 µm-thick Golgi-Cox stained sections, the method developed by Gibb and Kolb 1998 was specifically optimized for adult rat brains [33], which are considerably larger and structurally more robust than the brains of neonatal and early postnatal mice. To address these challenges, we found that cryostat sectioning provided better control over section quality and tissue preservation. Freezing the tissue stabilized the fragile neonatal brain, allowing the production of sharp, well-defined sections with minimal fragmentation. This approach eliminated the need for using vibratome and was particularly suitable for our low-cost and flexible workflow.

In our study, we show consistent staining of MSNs at PND7 and subsequent postnatal age groups in line with previous studies that used Golgi-Cox staining with unfixed adult brain tissue to avoid over impregnation and subsequent difficulties in completing quantitative analysis [16,26]. To determine whether more neurons could be visualized at PND1 and PND3, we tested PFA fixation prior to Golgi-Cox impregnation. While this approach initially appeared to increase the number of stained neurons, careful inspection at higher magnification revealed incomplete staining of dendritic branches, poorly stained dendritic spines, and undefined cell bodies compared to unfixed tissue. Previous reports highlighted the importance of the prior fixation step on the quality of Golgi-Cox staining on both adult and embryonic tissue [17,19,20]. The prior fixation step has been reported to significantly enhance staining quality and increase the number of labelled neurons in embryonic mouse brain tissue [19,20]. While brief, fixation of PND7 and 4-week old mouse brains before Golgi-Cox impregnation has resulted in excessively impregnated neurons in mouse cortex, preventing clearly visualizing the morphological details even in 100 μm thick sections [20]. These observations suggest that PFA fixation enhances labelling efficiency and tissue integrity in delicate neonatal brains; however, it may slightly compromise fine structural resolution at the level of individual cell soma. While our protocol demonstrates that successful staining and dendritic spine visualization can be achieved without PFA perfusion or prefixation, we recognize that this approach is also employed in commercial kits, such as the FD Rapid GolgiStain™ Kit (FD NeuroTechnologies, Inc, Columbia, MD, USA) which similarly recommend avoiding fixation prior to impregnation [34]. This method is particularly suitable for adult tissues, where the structural integrity of neurons can be preserved without fixation. However, studies attempting to adapt such kits for use in embryonic mouse brain tissue have reported that a prior aldehyde fixation step is necessary to stabilize fragile neural structures and improve staining consistency [19,20]. Interestingly, this requirement does not appear to extend to fetal sheep brain, where successful impregnation without prefixation has been reported [35], suggesting species- and stage-specific differences in tissue handling requirements. Our findings support the reliability of using an unfixed approach from PND1 through adulthood, yielding reproducible impregnation and clear morphological detail of dendritic structures. By employing a fully manual and cost-effective method, we provide an adaptable alternative that achieves results comparable to those obtained with commercial kits, without the need for specialized reagents or equipment.

Since Golgi-Cox staining selectively labels a random subset of neurons, it was challenging in this work to demarcate the anatomical boundaries between regions such as the striatum and surrounding structures in PND1 and PND3 brains by using Golgi-Cox staining only. This is attributed to incomplete structural development and maturation in the mouse neonatal brain. Therefore, it was necessary to combine Golgi-Cox with another staining protocol that allows clear visualization of neuronal architecture and precise demarcation of regional neuronal boundaries. Combining Golgi-Cox and Cresyl Violet was previously described in developmental neuroanatomical studies, to enable both detailed visualization of neuronal morphology and accurate demarcation of brain regions, which is particularly valuable in early postnatal stages when structural boundaries are not fully established [23,36]. This combined approach enabled clearer identification of brain region boundaries based on cytoarchitectural features, which are less distinct in mouse brains younger than PND7. The approach described in our work is compatible with both fixed and unfixed PND1 and PND3 brain slices that were impregnated before with Golgi-Cox.

One of the main drawbacks of the Golgi-Cox method is that it is a time-consuming technique. To address this, previous protocols have attempted to shorten the impregnation duration while maintaining high staining quality [27,29,33]. In our work, we aimed at staining subcortical structures that usually require a longer impregnation time compared to cortical neurons [37]. Considering the differences in tissue maturity, density, and fragility, we tested two different impregnation times to see if early neonatal brains, specifically, PND1 and PND3, which are less than half the size of adult brains could achieve adequate staining with a shorter protocol. However, our findings demonstrated that a 7-day impregnation period failed to produce staining quality of MSNs comparable to the standard 14-day protocol, indicating that a full 14-day impregnation is critical for optimal staining of neurons in the striatum, cortex, and hippocampus, regardless of brain size. This finding is in line with a previously published protocol [17] in which the impregnation period of 14 days was optimal for the staining of MSNs in the adult mouse brain. In contrast, another study [29] explored the reduced impregnation period on hippocampal neurons and found that staining cortical and hippocampal neurons was achievable after 36–48 h at 37 °C. Our findings indicate that a 14-day impregnation period enhances the consistency and quality of neuronal labeling in neonatal tissue.

Although this method was originally optimized for neonatal and early postnatal tissue, it has also been successfully applied to adult brain tissue. A key advantage over previously published protocols is the ability to collect sections post-cutting and temporarily store them in an appropriate buffer for later mounting, rather than requiring immediate mounting, making the protocol more convenient and practical. Our approach offers the added benefit of allowing brain sections to be stored at 4 °C for 3–7 days before slide mounting. To assess whether short-term storage of cryosections affects tissue quality, we compared adult brain sections that were mounted immediately after cryostat cutting with those stored in PBS at 4 °C for up to 7 days. Microscopic evaluation demonstrated that short-term post-sectioning storage is a viable option that enables greater flexibility during labor-intensive workflows, without compromising morphological detail or staining quality. Long-term freezing of whole brains prior to sectioning is commonly used in immunohistochemical protocols [38,39], it typically involves rapid freezing in liquid nitrogen or isopentane-dry ice mixtures using specialized molds. However, this approach is known to carry significant risks, including ice crystal formation, tissue shrinkage, and structural distortion—all of which may affect the delicate architecture of dendritic spines and neuronal morphology. In the context of Golgi-Cox staining, additional concerns include the potential for freezing-induced artifact precipitates that could interfere with the chromogenic development step. Future studies could further evaluate whether modified freezing protocols—such as pre-treatment with cryoprotectants followed by rapid freezing—can minimize tissue damage and expand storage options, particularly for delicate neonatal brain tissue.

Compared to commercially available Golgi staining kits [34], the protocol optimized in this study offers a significant advantage in terms of cost-effectiveness and flexibility. While commercial kits typically are expensive, our in-house protocol uses readily available cheap reagents. Furthermore, the flexibility in solution preparation and storage conditions, as well as the ability to adapt the protocol for different developmental stages, makes it a more practical choice for laboratories conducting high-throughput or longitudinal studies. In our experience, the staining quality and structural preservation achieved with our method are comparable to those obtained using commercial kits, especially in terms of spine clarity and dendritic morphology. This positions our protocol as a reliable, low-cost alternative suitable for detailed neuroanatomical studies across developmental time points.

## 4. Materials and Methods

### 4.1. Solution Preparations

#### 4.1.1. Golgi-Cox Solution Preparation

The Golgi-Cox Solution was prepared as previously described [17]. Solution A was prepared by adding 15 g potassium dichromate (K2Cr2O7; 05355, GCC, Clwyd, UK), stirred into 300 mL of warm distilled water (dH2O) to make a 5% (w/v) potassium dichromate solution. Solution B was prepared by adding 15 g of mercuric chloride (HgCl2; 04574, GCC, Clwyd, UK), stirred into 300 mL of hot dH2O to make a 5% (w/v) mercuric chloride solution. Solution C was prepared by adding 15 g of potassium chromate (K2CrO4, 05344, GCC, Clwyd, UK), stirred into 300 mL of cold dH2O to make a 5% (w/v) potassium chromate solution.

Solutions A and B were mixed, and 600 mL of dH2O was added to 240 mL of solution C. Solution A/B was then slowly poured into diluted solution C, with continuous stirring. The Golgi-Cox solution was mixed at room temperature until the characteristic red-yellow precipitate formed, typically within 10 min. Mixing continued for an additional 30 min before the solution was removed from the stirrer and kept in the dark for at least one hour prior to filtration. The Golgi-Cox solution was filtered before use and stored in the dark (at room temperature). This solution can be used for up to 1 month. All chemical preparations, including the mixing of Solutions A and B, were carried out in a fume hood, with laboratory staff wearing suitable personal protective equipment (PPE) to ensure safety.

#### 4.1.2. Cryoprotectant Solution Preparation

Cryoprotection is essential to protect cryosections, including the preservation of morphology and protecting frozen tissue from artifacts. We tested three cryoprotectant solutions: Cryoprotectant (1) was prepared as described previously [16] with some modifications. A 100 mL of a tissue-protectant solution is prepared by dissolving the following components: 25 g sucrose (C_12_H_22_O_11_; Labchem, Pittsburgh, PA, USA) and 15 mL glycerol (C_3_H_8_O_3_; Labchem, Pittsburgh, PA, USA). The final volume is then adjusted to 100 mL with PBS (pH 7.4). This volume is sufficient for five mouse brain samples. Cryoprotectant (2) was prepared as described previously [26], by dissolving 30 g sucrose (C_12_H_22_O_11_; Labchem, Pittsburgh, PA, USA) and 30 mL ethylene glycol (C_2_H_6_O_2_; Labchem, Pittsburgh, PA, USA). The final volume was adjusted to 100 mL by adding PBS (pH = 7.4). Cryoprotectant (3) was prepared by dissolving 25 g of sucrose in 100 mL of dH2O as described earlier [17]. Cryoprotectant solutions do not require protection from light prior to use.

#### 4.1.3. PFA Solution (4%) Preparation

PFA solution was prepared by mixing 18 g Di-sodium hydrogen phosphate dihydrate (Na2HPO4.2H2O, LOBA Chemie, Mumbai, India) and 9 g Sodium chloride (NaCl, Labchem, USA) to 1 L of dH2O, and the pH was adjusted to 7.4, then 40 g of PFA powder (Fisher Chemicals, Waltham, MA, USA) were added to the solution with heating kept below 65 °C and the pH maintained at 7.3 in fume hood.

#### 4.1.4. Cresyl Violet Solution Preparation

To prepare a stock solution, 100 mg of Cresyl Violet acetate powder (ChemScene LLC, Monmouth Junction, NJ, USA) was completely dissolved in 75 mL of warm dH2O (45 °C). To prepare working Cresyl Violet solution, 0.1 M sodium acetate solution was prepared by dissolving 8.2 g of sodium acetate anhydrous (CH3.COONa, LOBA Chemie, Mumbai, India) in 1000 mL of dH2O, and then acetic acid solution was prepared by dissolving 6 mL of acetic (glacial acid) in 1000 mL of dH2O. Later, 15 mL of solution 1 was mixed with 185 mL of solution 2. Then, 24 mL of the Cresyl Violet acetate was added to the previous mix of solutions.

#### 4.1.5. Gelatin-Coated Slide Preparation

To avoid detaching brain slices from microscopic slides during staining steps, slides used in this research were double subbed in 1% gelatin solution that was prepared by dissolving 10 g gelatin in 1000 mL of dH2O with constant stirring at 60 °C until the gelatin was dissolved. Then, 0.5 g chromium potassium sulfate was added to the solution and continuously stirred. At the end, the solution was filtered with filter paper. Clean plain slides were dipped in the rack into the solution and placed in an oven at 37 °C overnight; this step was repeated the next day. Slides were left to dry and stored in their original packages until use. Commercially available adhesive and positively charged slides were also used in this work without any notable issues.

### 4.2. Experimental Animals

Pregnant C57BL/6J mice were purchased and bred in the animal facility at Jordan University of Science and Technology (Irbid, Jordan). Offspring remained with their dams and were collected at various postnatal stages. A total of thirty-five mice were used, with five animals per age group (PND1, 3, 7, 14, 21, 30, and 3 months). Following weaning, animals were group-housed (3–5 per cage) under standard 12 h light/dark cycles, with unrestricted access to food and water. All experimental procedures were conducted in accordance with the guidelines of the Animal Care and Use Committee (ACUC) at Jordan University of Science and Technology.

### 4.3. Collecting Tissue and Cryoprotection

**Figure 9:**
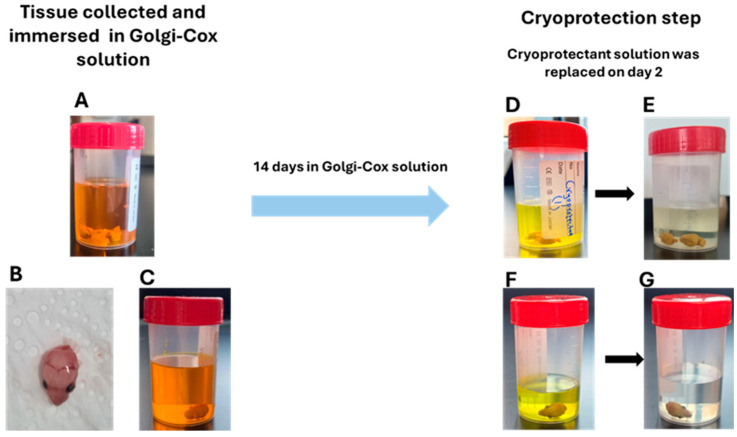
Golgi-Cox Impregnation. Dissected brains were hemisected and immersed in Golgi-Cox solution (**A**). Whole heads from PND1 and PND3 mice were collected (**B**) and also immersed in Golgi-Cox solution for 14 days (**C**). After 14 days, the brains were transferred to cryoprotectant solution and stored in the dark. After 24 h, the cryoprotectant solution appeared yellowish (**D**,**F**), so it was replaced to restore clarity, and the samples were kept for an additional 48 h (**E**,**G**).

Mice were euthanized by cervical dislocation, and brains were rapidly and carefully extracted using a spatula to minimize tissue damage. The delicate pia mater was gently removed using fine forceps as described in [40]. Brains were rinsed with normal saline to remove residual blood and enhance Golgi-Cox impregnation. To facilitate better solution penetration, each mouse brain was hemisected along the midsagittal plane into two equal halves using a razor blade, and both hemispheres were placed together in 20 mL of Golgi-Cox solution (Figure 9A). A minimum solution-to-tissue volume ratio of approximately 40:1 was maintained to ensure thorough impregnation and to facilitate optimal penetration and staining efficiency. The whole heads of PND1 and PND3 pups were collected to minimize the risk of damaging the delicate brain tissue during dissection and to facilitate handling and sectioning (Figure 9B). After removing the skin, the whole heads were immersed in Golgi-Cox solution (Figure 9C). Time required for impregnation: The total immersion time in the Golgi-Cox solution was 14 days as described in [16,17,33]. Initially, the tissue was incubated undisturbed for 24 h. The solution was then replaced on day 2 and subsequently refreshed every 1–3 days for 14 days. This 1–3-day replacement interval reflects optimization practices reported in the literature and was implemented to ensure staining consistency. To prevent potential chemical interactions with the heavy metal-based reagents used in the Golgi-Cox staining procedure, all tissue handling was performed using plastic instruments. Metal forceps were strictly avoided with Golgi-Cox, as they may compromise staining quality or lead to artifact formation. Tissues treated with Golgi-Cox solution should be protected from exposure to light and samples must be stored in a dark place.

Time required for impregnation of PND1 and PND3: Adult mouse brains, characterized by fully developed and denser neural tissue, require longer impregnation periods to ensure thorough penetration of the Golgi-Cox staining solution. In contrast, neonatal mouse brains are significantly smaller, both in size and weight, less mature, and more fragile. The neonatal tissues used in this study (PND1 and PND3) were less than half the size of adult brains. Since the P7 brain closely resembles the size and shape of an adult brain, the average brain weight for P7 mice was approximately 200–220 mg, while the brain weight for adult mice ranged from 380 to 400 mg [41]; tissue from P7, P14, P21, P30, and 3-month-old mice was impregnated in Golgi-Cox solution for 14 days. For neonatal tissue, we tested two different impregnation durations 7 days and 14 days—to determine optimal staining quality.

Cryoprotection step. On day 14, plastic forceps were used to remove the brains from the Golgi-Cox solution. The brains were briefly placed on filter paper to remove excess solution, then transferred into cryoprotectant solution and stored in the dark at room temperature for a minimum of three days. On day 2, the cryoprotectant solution appeared yellowish (see Figure 9D,F). To restore its clarity, the solution was replaced with fresh cryoprotectant (Figure 9E,G), and the brains were stored for an additional 48 h. Approximately 20–30 mL of cryoprotectant solution was used for each brain.

### 4.4. Cryosectioning Step

**Figure 10:**
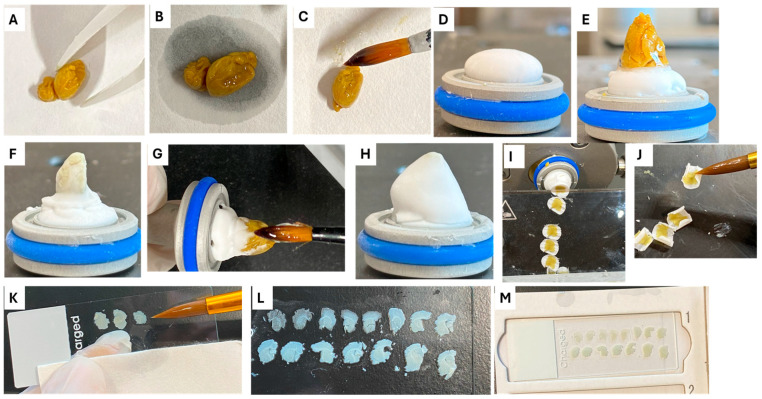
Cryosectioning step. The brain is removed from cryoprotectant solution with plastic forceps and placed on filter paper to remove excess fluids (**A**,**B**). A base of OCT medium is added to the sample holder and placed in the cryostat to freeze (**C**). A small amount of OCT medium is added to the surface of the brain using paint brush, and the sample is mounted on the sample holder with the rostral end above (**D**,**E**). The tissue was left to freeze for 20 min (**F**), and then a layer of OCT medium was added using paint brush (**G**). Brains were left in the cryostat for at least another 30 min until it is completely frozen (**H**). Chucks were mounted and cutting using low-profile blades was performed at 100 μm thickness in fast and continuous strokes. Anti roll glass plate is important during cutting to avoid rolling or curling of the sections (**I**). After cutting about 10 sections, the sections were retrieved by the tip of thick paint brush (**J**) and mounted immediately onto slides where a few drops of PBS had been applied to aid in smooth and accurate positioning and a piece of filter paper was placed along the lower edge of the slide to absorb excess PBS and minimize tissue displacement during mounting (**K**,**L**). Make sure that there are no air bubbles or solutions trapped between the section and the slide to prevent detaching of brain sections at later steps. Slides with mounted sections were kept drying at room temperature overnight in a dark place inside cardboard slide trays (**M**).

Neonatal and postnatal Golgi-impregnated brains are very delicate and can be easily disintegrated when cutting using a microtome [17]. Therefore, our technique used cryostats for tissue sectioning to ensure that the delicate neonatal and early postnatal brains will remain intact and integrated. MEV cryostat (SLEE medical GmbH, Mainz, Germany) was used. The chamber temperature of the cryostat should be set between −20 °C and −25 °C. Brains were gently removed from the plastic pot using plastic forceps and placed on filter paper to get rid of excess cryoprotectant (Figure 10A,B). A small amount of optimal cutting temperature (OCT) medium (Bio-Optica, Milan, Italy) was added to fill the gaps on the surface of the brain (Figure 10C). A base of OCT medium was added to the pre-cooled chuck (Figure 10D), and then the tissue was mounted directly onto the semi-frozen OCT with the rostral end of the brain directed upward (Figure 10E). Tissue was left to freeze for 20 min (Figure 10F), and then a layer of OCT medium was added using a paint brush (Figure 10G). Brains were left in the cryostat for at least another 30 min until completely frozen (Figure 10H). Chucks were mounted, and cutting using low profile blades, Leica 819 (Leica Biosystems, Nussloch, Germany) was performed at 100 μm thickness in fast and continuous strokes to minimize the time of blade contact and preserve the integrity of tissue as much as possible. Anti-roll glass plate is important during cutting to avoid excessive rolling or curling of the sections (Figure 10I). After cutting approximately 10 sections, the anti-roll plate was lifted, and the sections were gently retrieved using the tip of a thick paint brush (Figure 10J) and mounted immediately onto gelatin coated slides after adding a few drops of PBS to the slide to flatten the tissue and adjusting the position of the sections. Excess PBS (or cryoprotectant, if present) was carefully removed using a piece of filter paper, either by gently blotting or by placing the paper along the bottom edge of the slide as shown in (Figure 10K). This step ensured better adherence of the tissue to the slide and helped prevent folding or displacement (Figure 10L). After mounting, it is important to avoid putting any pressure on the mounted sections to avoid damaging the tissue. Air bubbles or trapped solutions between the section and the slide were excluded to prevent detaching of brain sections at later steps. Slides with mounted sections were kept drying at room temperature overnight in a dark place inside cardboard slide trays (Figure 10M). The sectioning method described in this paper does not require cryomolds or pre-freezing the tissue with precooled isopentane or liquid nitrogen. All slides and PBS used during section collection were kept at room temperature.

For adult (3-month-old) brains, it was feasible to collect tissue in PBS. Sections were placed in 50 mL plastic containers filled with PBS and kept at room temperature throughout the cutting process. Once sectioning was completed, the containers were stored at 4 °C for a period of 3–7 days prior to mounting. Tissue quality was regularly monitored by visual inspection; any signs of section curling, fragmentation, or degradation were considered indicators of compromised integrity, and such sections were deemed unsuitable for further analysis. Longer storage durations (more than 7 days) were not evaluated.

**Figure 11:**
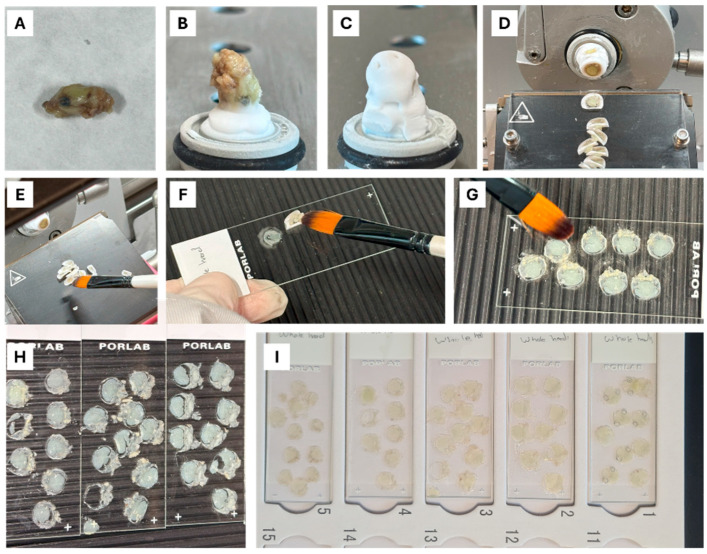
Neonatal brain tissue processing prior to staining. (**A**) Dissected neonatal mouse heads were removed and placed on filter paper to remove excess fluids. (**B**,**C**) Tissue mounted on sample holders with the rostral end downward, embedded in OCT medium, and frozen in the cryostat for at least 30 min. (**D**) Sectioning using a cryostat with an antiroll glass plate in place was performed with continuous cutting and fast strokes to avoid damaging or tearing the tissue. (**E**,**F**) Sections were transferred by the tip of paint brush with minimal mechanical stress to preserve tissue integrity and mounted into slides prepared with few drops of PBS. (**G**) Fine adjustments to the position of the tissue sections were made using a large paintbrush. (**H**) Final prepared slides ready for staining or imaging, demonstrating efficient and reproducible section collection. (**I**) Sections mounted onto slides were left to dry for at least 24 h in cardboard boxes.

For sectioning neonatal mouse heads, the same cryoprotection and cryostat protocols used for dissected brains (as outlined in Figure 10) were followed. Following dissection, the heads were placed on filter paper to absorb any excess moisture (Figure 11A). To minimize the loss of striatal tissue during the initial cuts, the heads were positioned with the rostral end facing downward, embedded in OCT medium, and left to freeze in the cryostat for at least 30 min (Figure 11B,C). This orientation helped preserve the striatum in the deeper sections with minimal tissue loss. Cryostat sectioning was performed using an anti-roll glass plate, with continuous and rapid cutting motions to prevent tissue damage or tearing (Figure 11D). The sections were carefully transferred using the tip of a paint brush to minimize mechanical stress and maintain tissue integrity and then mounted onto slides prepared with drops of PBS (Figure 11E,F). Fine positioning of the tissue sections was carried out using a paintbrush (Figure 11G,H), after which the sections were allowed to air-dry in cardboard boxes for a minimum of 24 h (Figure 11I).

### 4.5. Staining Procedure

#### 4.5.1. Preparation of Development Solution

To prepare 28% ammonia solution from 35% ammonia solution (NH_4_OH, 35%; Fisher Scientific, Loughborough, UK), 50 mL of dH2O were added to 200 mL of 35% ammonia according to the formula C1V1 = C2V2. Ammonium hydroxide must always be handled in the fume hood. Development step: Slides were washed with dH2O for 2 min each. Then, slides were incubated in the freshly prepared 28% ammonium hydroxide solution for 10 min in the dark in a fume hood. After development, slides were rinsed twice with dH2O for 5 min each to remove the excess Golgi-Cox solution.

#### 4.5.2. Dehydration and Mounting Step

Sections were dehydrated by processing the slides through 70%, 95%, and 100% (twice) ethanol for 5 min each. Then sections were cleared with xylene twice for 5 min each. DPX Mounting Medium (TECHNO PHARMACHEM, Haryana, India) was used to mount the slides, which were kept in black boxes in a dark room for at least 5 days until microscopic examination. To remove coverslips, soak slides in xylene until coverslips detach. Rinse sections in two changes of fresh xylene, followed by descending ethanol concentrations (absolute, 95%, 70%), and finally in dH2O before proceeding to counterstaining.

#### 4.5.3. Combined Golgi-Cox and Nissl Staining for Neonatal Brains (PND1 and PND3)

Golgi-Cox and Nissl staining were combined previously to precisely define the anatomical boundaries of the mouse brain and visualize overall cytoarchitecture [36] In this study, we combined Golgi-Cox and Cresyl Violet staining in PND1 and PND3 neonatal brains since it was difficult to clearly delineate the striatal boundaries at this developmental stage. Compatibility Considerations: To ensure compatibility between Golgi-Cox staining and subsequent Cresyl Violet counterstaining, tissue sections were fully impregnated and developed prior to Nissl staining. Golgi-Cox staining must be performed first, as it relies on heavy metals and chromate-based precipitation, which can be disrupted by subsequent chemical treatments. Additionally, due to the relatively thick sections (100 µm), sufficient immersion time in Cresyl Violet solution was ensured to allow adequate penetration and uniform staining. Throughout the procedure, sections were never allowed to dry, as drying can compromise staining quality and tissue integrity. Both Golgi-Cox and Cresyl Violet signals should be clearly visible under standard light microscopy, enabling complementary visualization of neuronal morphology and anatomical features.

Cresyl Violet staining. Following completion of the development step described in Section 4.5.1, sections were processed for Nissl staining. Sections were first dehydrated through an ascending ethanol series incubated as follows: 5 min in 70% alcohol, 5 min in 95% alcohol, and 5 min in 100% alcohol. Subsequently, sections were incubated for 20 min in a 1:1 mixture of chloroform and absolute ethanol to facilitate clearer staining. Following this, the tissue was rehydrated by immersion in 95% alcohol for 5 min, 70% alcohol for 5 min in 70% alcohol, and dH2O for 5 min. Rehydrated sections were then incubated for 10–15 min in Cresyl Violet solution prepared as described in Section 4.1.4. After staining, sections were rinsed for 5 min in dH2O to remove excess dye. Sections then underwent dehydration through increasing concentrations of alcohol ladder as follows: 70% (2 min), 95% (5 min), and 100% (5 min). Tissue clearing step was performed by immersing the sections in xylene for 5 min, followed by cover slipping with DPX mounting medium. All steps involving organic solvents (ethanol, chloroform, xylene) were carried out under a fume hood, and appropriate personal protective equipment (PPE) was worn throughout the procedure to ensure laboratory safety.

### 4.6. Image Acquisition

A light microscope (OPTIKA, Ponteranica, Italy) equipped with a digital camera (OPTIKA, Ponteranica, Italy) and a computer was used to examine and take images of the Golgi-stained slides. Images were captured at different magnifications: 100×, 400×, and 1000× using the Optika PROVIEW software (version 4.8; Ponteranica, Italy). Imaging and qualitative analysis were mainly focused on MSNs in the mouse striatum with additional imaging performed in the cortex and hippocampus for comparative purposes. Coronal sections were selected from defined anatomical levels according to the Mouse Brain Atlas, Reference atlas version 1 (2008) available at https://mouse.brain-map.org (accessed on 22 May 2025). Specifically, images of the cortex and striatum were taken from sections corresponding to Bregma levels +1.6 mm to −0.05 mm, while hippocampal images were acquired from Bregma −1.3 mm to −2.0 mm. For each brain region of interest, 3–5 well-impregnated sections per animal were selected. From each section, 2–3 non-overlapping fields were imaged at 40×, 400×, and 1000× magnifications. Images were used to qualitatively evaluate section integrity, dendritic structure, and spine clarity, across developmental stages.

### 4.7. Structural Preservation Analysis

To assess the quality and structural integrity of Golgi-Cox stained brain tissue across various developmental stages, randomly selected sections from each experimental group were examined using light microscopy. Observations were conducted at both low (100×) and high (400× and 1000×) magnifications. Evaluation criteria included uniform and consistent staining, intact section morphology, absence of folding, tearing, or cracking, clear cortical layer organization, and the ability to identify key brain regions such as the striatum, hippocampus, and cortex. At higher magnification, neurons were assessed for well-defined cell bodies, continuous and clearly stained dendritic branches, and minimal fragmentation. Dendritic spines were evaluated based on their clear distinction from dendritic shafts and minimal background interference, enabling accurate classification into morphological types (thin, stubby, and mushroom) as described in [11,17].

## 5. Conclusions

Together, the methodological choices implemented in this study—specifically, the use of cryostat sectioning and unfixed tissue—offer several advantages over previously published protocols. We provide a simplified and cost-effective method for clearly and efficiently visualizing MSNs in the brains of neonatal and early postnatal mice under light microscopy, without the need for prior PFA fixation or reliance on commercial kits or specialized equipment. These adaptations improved tissue preservation, enhanced image clarity, and streamlined the workflow, thereby expanding the applicability of Golgi-Cox staining in developmental neurobiology. Importantly, the protocol remains effective across developmental stages, from neonatal to adult brains, reinforcing its value as a versatile and accessible tool for studying neuronal morphology.

## Data Availability

No new data were created.

## Abbreviations

The following abbreviations are used in this manuscript:

MSNs	Medium Spiny Neurons
PND	Postnatal day
PFA	Paraformaldehyde
PBS	Phosphate buffered Saline
OCT	Optimal cutting temperature
NDDs	Neurodevelopmental disorders
dH2O	Distilled water
